# Quantitative adverse outcome pathway (qAOP) models for toxicity prediction

**DOI:** 10.1007/s00204-020-02774-7

**Published:** 2020-05-18

**Authors:** Nicoleta Spinu, Mark T. D. Cronin, Steven J. Enoch, Judith C. Madden, Andrew P. Worth

**Affiliations:** 1 School of Pharmacy and Biomolecular Sciences, Liverpool John Moores University, Byrom Street, Liverpool, L3 3AF UK; 2 European Commission, Joint Research Centre (JRC), Ispra, Italy

**Keywords:** Predictive toxicology, Quantitative adverse outcome pathway (qAOP), Computational approach, Bayesian network, Response-response relationship, Key event relationship

## Abstract

The quantitative adverse outcome pathway (qAOP) concept is gaining interest due to its potential regulatory applications in chemical risk assessment. Even though an increasing number of qAOP models are being proposed as computational predictive tools, there is no framework to guide their development and assessment. As such, the objectives of this review were to: (i) analyse the definitions of qAOPs published in the scientific literature, (ii) define a set of common features of existing qAOP models derived from the published definitions, and (iii) identify and assess the existing published qAOP models and associated software tools. As a result, five probabilistic qAOPs and ten mechanistic qAOPs were evaluated against the common features. The review offers an overview of how the qAOP concept has advanced and how it can aid toxicity assessment in the future. Further efforts are required to achieve validation, harmonisation and regulatory acceptance of qAOP models.

**Electronic supplementary material:**

The online version of this article (10.1007/s00204-020-02774-7) contains supplementary material, which is available to authorized users.

## Introduction

Since its establishment in 2010 (Ankley et al. 2010), the adverse outcome pathway (AOP) framework aimed to enhance efficiency and transparency in chemical safety assessment (OECD 2018). Recent progress in the development of AOPs covers a spectrum of novel endpoints and chemicals/categories including nanoparticles and other classes of stressors, e.g. microplastics and radiation (Chauhan et al. 2019; Jeong and Choi 2019; Jeong et al. 2018). Furthermore, new ways of deriving AOPs have been proposed such as data mining, deep learning or a combination of machine learning techniques (Carvaillo et al. 2019; Jeong et al. 2019; Rugard et al. 2020).

In addition to the increasing numbers of linear (qualitative) AOPs, AOP networks are being extensively applied and have considerable value. An AOP network is defined as a set of linear AOPs sharing common events and, therefore, representing a better depiction of biological processes (Knapen et al. 2018; Villeneuve et al. 2018). Examples of AOP network applications include: mapping chemicals to linear AOPs to identify common interactions (Aguayo-Orozco et al. 2019); understanding the mechanistic pathways leading to mitochondrial dysfunction (Dreier et al. 2019); identification of common key events (KEs) for chemical screening and integrated testing strategy for developmental neurotoxicity (Li et al. 2019); chemical assessment using biologically based testing batteries (Angrish et al. 2017); and the development of an exploratory AOP database to derive “putative” AOPs (Pittman et al. 2018). Moreover, progress has been made with regard to the use of topological features in the network, such as the degree to which the most common/highly connected paths within an AOP network can be identified (Pollesch et al. 2019; Spinu et al. 2019). Additionally, many molecular initiating events (MIEs) have been thoroughly modelled in silico due to their ability to describe the interaction between the stressor and the biological receptor at the molecular level that induces adverse effects (Allen et al. 2016). In silico models of MIEs are represented by 2-D and 3-D structural alerts and (Quantitative) Structure–Activity Relationships (Allen et al. 2019; Cronin and Richarz 2017; Mellor et al. 2016) and have been incorporated in mechanistically based toxicokinetic/toxicodynamic models that evaluate exposure–response relationships (Gao et al. 2019; MacKay et al. 2013).

Formerly, various types of AOPs were distinguished from qualitative to semi-quantitative and quantitative AOPs (qAOPs) (Perkins et al. 2015; Villeneuve et al. 2014). While qualitative AOPs can be used to guide chemical decision-making during the development of novel compounds including integration of diverse lines of evidence, prioritisation of testing strategies and screening of chemicals, design and development of fit-for-purpose assays, qAOP models can be seen as tools for quantitative risk assessment of chemicals (Carusi et al. 2018; Coady et al. 2019; Villeneuve et al. 2014). Hence, each type of AOP has potential utility in chemical risk assessment (Hecker and LaLone 2019). The concept of a qAOP as a predictive computational model is gaining interest due to its ability to address the question of how much perturbation, at any of the upstream KEs, and under what conditions, the adverse outcome (AO) is likely to occur (Conolly et al. 2017; Patlewicz et al. 2015). A qAOP helps to define the biological tipping point(s) along the pathway, and the probability or magnitude with which those tipping points are exceeded (Conolly et al. 2017; LaLone et al. 2017). Importantly, several international workshops have identified critical aspects in developing a qAOP model including the quantification of key event relationships (KERs), data availability, defining the threshold for inducing an effect, incorporation of modulating factors (e.g. genetic predisposition, previous exposures), establishment of mathematical rules for the KERs, parametrisation of non-linear models, and model validation and implementation (Kleinstreuer et al. 2016; Leist et al. 2017; Wittwehr et al. 2017). The extent to which these challenges are addressed by available qAOP models is not covered by the scientific literature. On the other hand, whilst knowledge is being acquired and systematically captured, there is no official guidance providing a coherent and all-encompassing framework for the development and assessment of a qAOP model. The existing guidance, developed by the Organisation for Economic Cooperation and Development (OECD), explains how to build evidence for an AOP and this highlights the importance of the quantitative understanding of the KER as a criterion in the assessment of the overall confidence of an AOP (OECD 2018). In addition, the OECD guidance on the use of AOPs in the development of Integrated Approaches to Testing and Assessment (IATA) states that a qAOP can help to target a KE and select the appropriate assays for test guideline development or refinement to predict the AO (OECD 2016).

## Focus of this review

The aim of this review was to evaluate the progress made in the qAOP concept in chemical safety assessment. The specific objectives were: to analyse published definitions of qAOPs in the scientific literature and formulate a set of common features of a qAOP model; and to assess the types of qAOP models based on the identified features that utilise probabilistic and mechanistic approaches, as well as methods and software tools used for modelling by screening relevant scientific literature in the Web of Science, Pubmed and Google Scholar databases published prior to October 2019.

## Computational modelling in the context of quantitative adverse outcome pathways

The OECD Guidance document on the use of AOPs in IATA (OECD 2016) defines a qAOP as “an assembly of KEs supported by descriptions of how the KEs can be measured and the accuracy and precision with which the measurements are made along with KERs supported by quantitative understanding of what magnitude and/or duration of change in the upstream KE is needed to evoke some magnitude of change in the downstream KE”. Despite this clear definition, the meaning of qAOPs has often been interpreted differently, with various definitions given and, as a result, varying expectations of the scientific community. Screening the scientific literature for the Medical Subject Headings (MeSH) term “quantitative Adverse Outcome Pathways”, 23 publications were found which refer to the concept of qAOP (Supplementary Information Table S1). The identified definitions were retrieved and analysed individually to identify and map a series of common features that the authors considered essential for the development of a qAOP model. Thus, a list of five common features for qAOP models was formulated encompassing the expectations of the scientific community (Table 1). These features help to understand how the modelling of a qAOP has been approached as well as opportunities for improving the modelling process. Related to the common features, a set of criteria were identified and used to characterise qAOP models published in the scientific literature (Tables 2, 3, 4).

**Table 1:** Common features of qAOP models in the scientific literature

Common feature	Description	Criteria^a^
Problem formulation	• A qAOP should answer a well-defined question relevant to the AO of interest • The purpose of the model dictates how much mechanistic understanding is required, and the way a qAOP should be developed, validated and used	• Question addressed and/or purpose of modelling • AO studied
Mechanistic knowledge and associated data	• The OECD AOP-Wiki can support the development of a qAOP model to predict an endpoint of interest. Empirical data for model parametrisation, fitting and/or testing can be obtained from the description of KERs published in the AOP-Wiki • Whilst for quantification it is recommended to start with linear AOPs, it should not impede quantification of networks or highly connected KEs/KERs within an AOP network • A qAOP model relies heavily on data: not only bioactivity of a compound/mixtures but also, measurements of effects at relevant doses/concentrations and appropriate time scales including physicochemical properties and molecular descriptors. Data may come from a range of in vivo and in vitro studies specifically designed to test an AOP as a hypothesis and/or retrieved from a variety of sources to assist with this process • Both adjacent and non-adjacent KEs paired as upstream–downstream in a KER should be quantified even though each of them impacts differently on the modelling process, e.g. in the context of Bayesian network modelling. Adjacency refers to whether there are other KEs positioned in between of the linear construction of an AOP or not • Different biological level of organisations should be quantified if this is relevant to the AO of interest and available data allowed	• Presence of the AOP in the OECD AOP-Wiki • Type of AOP: linear or network • Type of chemical model applied to (single chemical(s)/mixtures) • Type of data: in vivo, in vitro, in silico and/or other variables • Dose/concentration–responses • (D/C–R) and time–responses (T–R) • Adjacency of KERs: adjacency and non-adjacency • Biological levels: cellular, tissue, organ, organism, population
Quantitative approach	• The modelling approaches can vary from being probabilistic to deterministic • The mathematical expression can take various forms including linear regressions and ordinary differential equations resulting in different graphical shapes, e.g. linear, sigmoidal, Gaussian-type plots	• Type of quantitative approach
Regulatory applicability	• A qAOP model should imply various applications to regulatory decision-making and acceptance	• Human health/ecological risk assessment
Additional considerations	• These considerations can influence the regulatory approval, reduce the uncertainties, and extend the applicability domain of the predictions of a qAOP model • It is not mandatory that the test methods used (models and measured endpoints) are adopted/validated following national/international guidelines. However, they should be performed in a quality-controlled environment where relevance of the model is proved based on scientific rationale and reproducibility of data • Even though none of the definitions identified referred to uncertainty and sensitivity analysis, this aspect should be considered as well for its value in validating the predictions of a qAOP model while giving confidence in its further applications	• Cross species extrapolation • Modulating factors • Positive/negative feedback loops • Compensatory mechanisms • Test method adopted/validated • Kinetics • Exposure assessment • Uncertainty evaluation • Sensitivity analysis • Availability: open access or not

^a^The criteria were used to characterise available qAOP models

**Table 2:** Characterisation of five probabilistic models that use the Bayesian network approach and an AOP construct

Model purpose	Adverse outcome	Mechanistic knowledge and associated data								Quantitative approach	Regulatory applicability	References
		OECD AOP-Wiki ^a^	Type of AOP^d^	Type of chemical model applied to	Data type	Adjacent KERs	Biological level(s)	D/C–R^e^	T–R^f^			
The risk posed by pesticides and environmental stressors to population size of Chinook salmon	Alteration of population dynamics	No^b^	AOPN	Mixtures	In vitro experimental data, literature data, AOP construction, environmental factors, population characteristics	√	Molecular, cellular, organ, organism, population	√	√	Bayesian Network-Relative Risk type of model	Ecological risk assessment	Chu (2018)
Effects on reproduction of *Lemna minor* (duckweed)	Reduced number of fronds	AOP ID 245	LAOP	Single chemical	In vitro experimental data, AOP construction	√	Molecular, cellular, organism	√	–	Bayesian network type of model (discrete states as three intervals)	Ecological risk assessment	Moe et al. (2018)
Toxicity of silver nanoparticles, linking MIE to the AO	Reproduction failure	AOP ID 207	LAOP	Nanoparticles	In vitro experimental data, literature data, AOP construction	√	Molecular, cellular, organ, organism	√	√	Bayesian network type of model (discrete states as yes/no, and decrease/stable/increase), Boostrapping	Ecological risk assessment	Jeong et al. (2018)
Occurrence of steatosis under different chemical exposures	Hepatic steatosis	No^c^	AOPN	Mixtures	Expert judgment, literature data, AOP construction	√	Molecular, cellular, tissue, organ	√	–	Bayesian network type of model (discrete states as active or inactive)	Human health risk assessment	Burgoon et al. (2020) and Perkins et al. (2019a)
Comparison between probabilistic and mechanistic approaches	Nephron attrition leading to chronic kidney disease	AOP ID 284	LAOP	Single chemical	In vitro experimental data on human RPTEC/TERT1 cells, AOP construction	√	Molecular, cellular, tissue, organ	√	√	Dynamic Bayesian network type of model	Human health risk assessment	Zgheib et al. (2019)^g^

^a^Numbers represent the indices (XXX) of the AOP in the AOP-Wiki available at https://aopwiki.org/aops/XXX

^b^Model follows an AOP structure, the MIE (ID 12) can be found in the AOP-Wiki, however the AOP itself is not yet published

^c^Model is included in the AOPXplorer tool (https://apps.cytoscape.org/apps/aopxplorer) as it follows the structure of an AOP network

^d^Linear AOP (LAOP), AOP Network (AOPN)

^e^Dose/Concentration–Response (D/C–R)

^f^Time–Response (T–R) describing the time-course behaviour

^g^Model represents a combination of both probabilistic and mechanistic approaches

**Table 3:** Characterisation of ten mechanistic qAOPs

Model purpose	Adverse outcome	Mechanistic knowledge and associated data								Quantitative approach	Regulatory applicability	References
		OECD AOP-Wiki^a^	Type of AOP^b^	Type of chemical model applied to	Data type	Adjacent KERs	Biological level(s)	D/C–R^c^	T–R^d^			
Association of MIE to AO at higher level of biological organisations	Increased frequency of spontaneous tail contractions	No	LAOP	Single chemical	In vivo experimental data	√	Molecular, tissue, organ	√	√	Statistical analysis	Ecological risk assessment	Yozzo et al. (2013)
Mechanism of CuO engineered nanoparticles toxicity	Mortality	No	LAOP	Nanoparticles	In vitro experimental data	√	Molecular, cellular, organ, organism	√	√	Linear regression, one-compartment toxicokinetic model	Ecological risk assessment	Muller et al. (2015)
Development of a qAOP network	Egg production	No	AOPN	Single chemical	In vitro and in vivo experimental data	√	Molecular, cellular, tissue, organ, individual	√	√	Statistical analysis	Ecological risk assessment	Margiotta-Casaluci et al. (2016)
Development of a qAOP and potential applications	Population declining trajectory (reproductive dysfunction)	AOP ID 25	LAOP	Single chemical	Empirical data	√	Molecular, cellular, tissue, organ, individual, population	√	√	A mechanistic model, a compartment model, a statistical model, a density-dependent population matrix model	Ecological risk assessment	Conolly et al. (2017)
Development of a qAOP on developmental neurotoxicity	Brain malformation	AOP ID 42	LAOP	Single chemical	In vivo experimental data	√^f^	Molecular, cellular, tissue, organ	√	√	Mathematical equations (exponential regression)	Human risk assessment	Hassan et al. (2017)
Development of a cross-species qAOP	Mortality increase, population declining trajectory	AOP ID 150	LAOP	Mixtures	In vitro experimental data on COS-7 cells	√^f^	Molecular, organism, population	√	–	Linear regression, statistical analysis	Ecological risk assessment	Doering et al. (2018)
Simulation of the mechanism of toxicity	Abnormalities at facial primordia branchial arches	No	LAOP	Single chemicals	In vitro experimental data, in vivo and in silico data	√	Molecular, cellular, tissue, organ	√	√	Multistage dose–response model, Bayesian analysis	Ecological risk assessment	Battistoni et al. (2019)
Define the taxonomic domain of applicability of an existing qAOP	Decreased fecundity	AOP ID 25	LAOP	Single chemical	In vivo experimental data	√	Cellular, tissue, organ, individual	√	√	Regression, statistical analysis	Ecological risk assessment	Doering et al. (2019)
Quantification of qKERs with available data in a modular manner	Decrease in population; Impairment of memory and learning	AOPs IDs 25 and 48	LAOP	Single chemicals	Empirical data	√^f^		√	–	Linear regression (response-response function)	Screening or prioritisation	Foran et al. (2019)
Comparison between probabilistic and mechanistic approaches	Nephron attrition leading to chronic kidney disease	AOP ID 284	LAOP	Single chemicals	In vitro experimental data on human RPTEC/TERT1 cells, AOP construction	√	Molecular, cellular, tissue, organ	√	√	Empirical dose–response model, systems biology model	Human health risk assessment	Zgheib et al. (2019)^e^

^a^Numbers represent the indices (XXX) of the AOP in the AOP-Wiki available at https://aopwiki.org/aops/XXX

^b^Linear AOP (LAOP), AOP Network (AOPN)

^c^Dose/Concentration–Response (D/C–R)

^d^Time–Response (T–R) describing the time-course behaviour

^e^Model represents a combination of both probabilistic and mechanistic approaches

^f^Non-adjacent KERs were modelled as well

**Table 4:** Characterisation of the available qAOP models based on the additional considerations listed in Table 1

References	Cross species extrapolation	Modulating factors	Feedback loops	Compensatory mechanisms	Test method adopted/validated	Kinetics	Exposure assessment	Uncertainty evaluation	Sensitivity analysis	Publicly available
Chu (2018)	–	√	–	–	–	–	√	√	√	√
Moe et al. (2018)	–	––	–	–	√^a^	–	–	√	√	√
Jeong et al. (2018)	–	–	–	–	–	√	–	–	√	√
Burgoon et al. (2020) and Perkins et al. (2019a)	–	–	–	–	–	–	–	–	√	√
Zgheib et al. (2019)	–	–	–	–	–	–	–	√	√	√
Yozzo et al. (2013)	–	–	–	–	–	–	–	–	–	–
Muller et al. (2015)	–	–	–	–	–	√	–	–	–	√
Margiotta-Casaluci et al. (2016)	–	–	√	–	–	√	–	–	√	–
Conolly et al. (2017)	–	–	√	√	–	√	–	√	–	–
Hassan et al. (2017)	–	–	√	√	–	√	√	√	–	√
Doering et al. (2018)	√	–	–	–	–	–	–	√	–	√
Battistoni et al. (2019)	–	√	√	–	–	–	√	√	–	–
Doering et al. (2019)	√	–	–	–	–	√	–	√	√	–
Foran et al. (2019)	–	–	–	–	–	–	–	–	–	–
Zgheib et al. (2019)	–	–	–	–	–	–	–	√	√	√

^a^The in vitro measurements were conducted on a plant recognised in the OECD test guidelines for toxicity testing of the endpoint

Three conceptual classes of qAOPs have been suggested:

1. Semi-quantitative/quantitative weight-of-evidence (semi-q/qWoE) qAOPs. These utilise quantitative weighting and numerical assessments of multiple lines of evidence to rank the confidence in KERs for further quantification (Gust et al. 2016; Perkins et al. 2019b). For example, to calculate the quantitative confidence scoring of KERs of a linear AOP, Bradford Hill considerations (biological plausibility, essentiality, dose–response concordance, consistency, and analogy) were proposed in a conceptual method by Becker et al. (2017), while Collier et al. (2016) additionally used metrics related to data quality for the KEs.
2. Probabilistic qAOPs and qAOP networks. These are computational models that incorporate statistical or probabilistic approaches such as Bayesian networks covering few events or an entire AOP to build predictive relationships between MIEs and/or KEs linked to apical outcomes (Gust et al. 2016; Perkins et al. 2019b).
3. Mechanistic qAOPs and qAOP networks. These are computational models defined as deterministic models where mathematical functions of the MIE, KE and KER can be used to predict the likelihood that a later event or AO would occur based on changes in an earlier event given specified initial conditions (Gust et al. 2016; Perkins et al. 2019b).

**Fig. 1:**
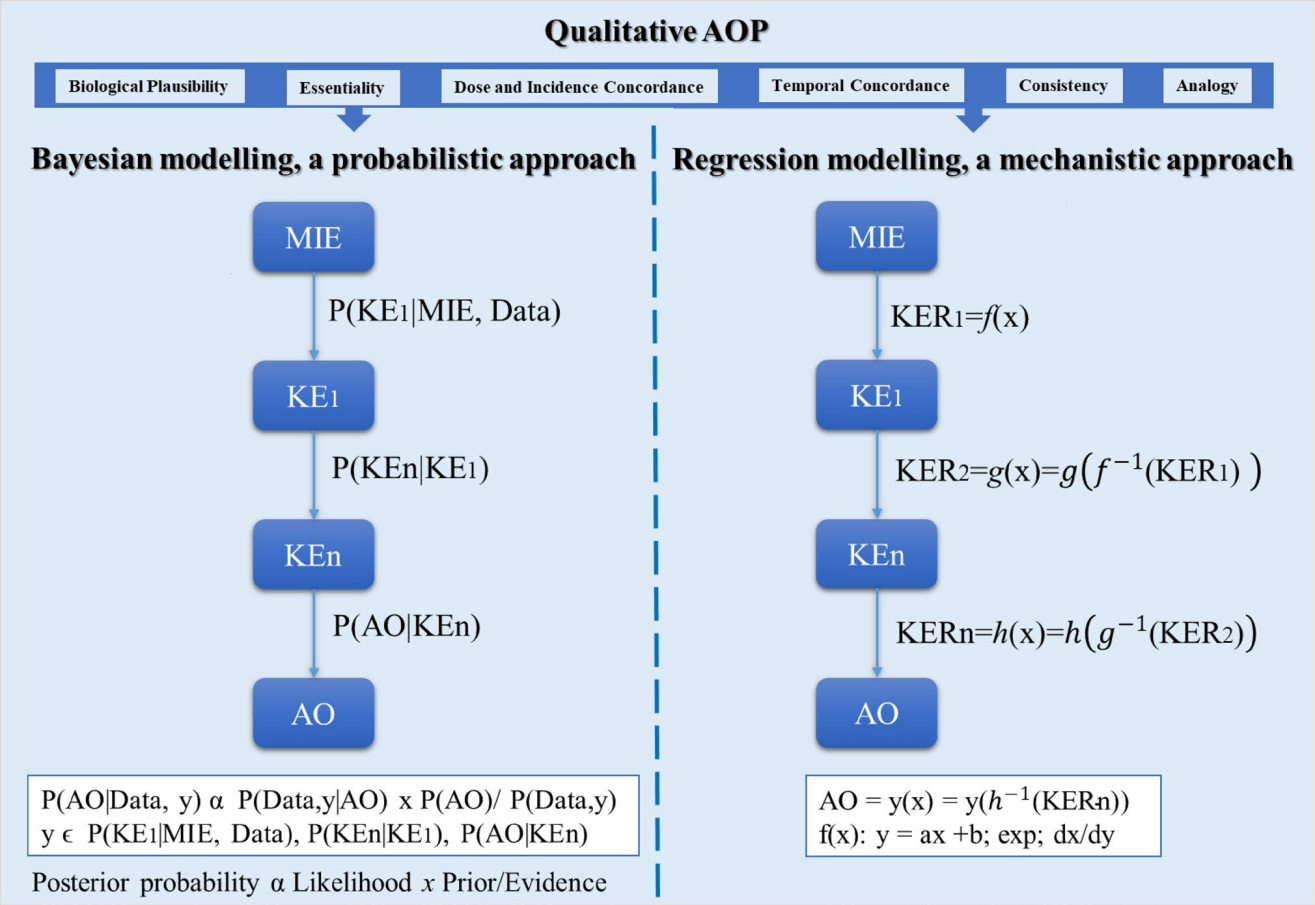
Conceptual representation of available types of qAOP models. Qualitative AOPs have an informative role for prioritisation and computational modelling of the AO of interest and can additionally be quantified by a weight-of-evidence. A common approach to probabilistic modelling relies on the use of Bayes theorem as described below. Mechanistic qAOP models utilise mathematical functions including linear regressions

The definitions of the qAOP concept as identified in the scientific literature support all these types of qAOP models, with only a small proportion (fewer than 10%) referring to semi-q/qWoE qAOPs, and approximately 25% to probabilistic qAOPs while all papers referred to mechanistic qAOPs. Therefore, whilst the first type of qAOP can be regarded as an extension of a qualitative AOP with empirical data, the second and third types of qAOP are mathematical models, distinguished according to the type of modelling approach. Thus, the first type of qAOP is conceptually different to the second and third. An opportunity is to make use of semi-q/qWoE qAOPs to develop predictive models based on probabilistic or mechanistic approaches as graphically presented in Fig. 1.

## Overview of probabilistic quantitative linear AOPs and AOP networks

Bayesian networks use a directed acyclic graph (DAG) to represent conditional probability relationships. Each node in the network corresponds to a KE or additional variable, e.g. physicochemical properties, while edges show the conditional dependencies between two KEs that form a KER. In other words, the Bayesian network uses conditional probability tables (CPTs) for each KE (node) to determine the probability of activity for parent and child nodes, i.e. an upstream KE leading to a downstream KE based on the Bayes’ rule, which is the unique mathematical equation for this type of modelling. Whilst the choice of KEs in the DAG is informed by the structure of the AOP, a Bayesian network can be entirely data-driven and may, or may not, be consistent with the topology of the AOP. Therefore, the Bayesian network approach has other applications in predictive toxicology in addition to qAOP development. These include: identification of the best biomarkers to characterise chemical exposure using dose–response analysis to determine the points of departure (Hack et al. 2010); development of an efficient testing strategy (Jaworska et al. 2015); classification of chemicals based on a mode of action (Carriger et al. 2016); classification of the cellular effects of nanoparticles (Furxhi et al. 2019); and prediction of the severity level of drug induced liver injury (Williams et al. 2019).

Currently, five qAOP models have been identified that follow the Bayesian approach and were assessed in terms of the common features including the additional considerations (Tables 2, 4).

### Problem formulation

A variety of purposes can be recognised across the available probabilistic qAOPs models. The AOs covered by these models include organ failure or ecotoxicological population level endpoints.

### Mechanistic knowledge and associated data

Three of the probabilistic qAOPs are available in the AOP-Wiki (AOPs IDs 207, 245, 284). Two probabilistic qAOPs utilised AOP networks. The qAOP of Moe et al. (2018) included a linear AOP with KEs represented by multiple measurements, e.g. oxidative phosphorylation and formation of reactive oxygen species to describe the first KE. All probabilistic qAOP models incorporated various types of data including experimentally derived and/or judgement-based results. Moe et al. (2018) and Jeong et al. (2018) quantified AOPs of interest using experimental data, while Chu (2018) conducted specific experiments and Perkins et al. (2019a) used a combination of in vitro data and expert judgment. Importantly, probabilistic approaches are flexible and can estimate predictions for both single chemicals and mixtures more easily than mechanistic approaches, e.g. binary assumption of a state of a KE. As a result, Perkins et al. (2019a) quantified liver steatosis caused by both individual, and a mixture of, chemicals. Likewise, Chu (2018) analysed the exposure to single organophosphate pesticides and binary and tertiary mixtures (synergistic effect). However, not all of the probabilistic qAOPs assessed this aspect, i.e. mixture vs individual chemicals. For example, Moe et al. (2018) quantified the linkage between exposure to 3,5-dichlorophenol to reduced number of fronds in the aquatic plant *Lemna minor*. Interestingly, nanoparticles were assessed in addition to single (small) organic compounds. As such, Jeong et al. (2018) quantified the reproductive toxicity of silver nanoparticles induced via oxidative stress in the nematode *Caenorhabditis elegans*. All probabilistic qAOPs made an attempt to link molecular/cellular effects to organ effects through adjacent KERs. However, not all probabilistic qAOP models accounted for dose and time responses. Whilst all included dose responses, only Chu (2018), Jeong et al. (2018) and Perkins et al. (2019a) made time predictions.

### Quantitative approaches

Moe et al. (2018) formulated CPTs based on the count of observations and statistical analysis. Comparing these two CPTs, those based on the count of observations gave more accurate predictions at high and low stressor concentrations, while CPTs based on statistical models gave better predictions at intermediate stressor concentrations. When no information is available, the probability of activation can be set at 50%, for example, the qAOP model developed by Perkins et al. (2019a). Another important aspect is the type of variables used to define the nodes, in discrete or continuous forms. Most qAOP models defined the nodes as discrete states: intervals (Moe et al. 2018), yes/no and decrease/stable/increase (Jeong et al. 2018), active/inactive (Perkins et al. 2019a) and categories/groups of intervals or periods of time (Chu 2018). Depending on its scope, the Bayesian network can have different outputs: the probability of a compound being active at a given concentration (Perkins et al. 2019a); the prediction of responses of each KE at different concentrations (Moe et al. 2018); the calculation of a relative risk (Chu 2018); or the analysis of causal relationships between KEs (Jeong et al. 2018).

### Regulatory applicability

Two of the qAOP models are applicable in human health risk assessment (Burgoon et al. 2020; Perkins et al. 2019a; Zgheib et al. 2019), two qAOP models in ecological risk assessment (Chu 2018; Moe et al. 2018) and a single qAOP model in nanoparticle risk assessment (Jeong et al. 2018).

### Additional considerations

None of the qAOP models included kinetic considerations, non-adjacent KERs, details about compensatory mechanisms or feedback loops. However, the qAOP model developed by Chu (2018) considered modulating factors such as environmental conditions, e.g. temperature and dissolved oxygen. Furthermore, the qAOP of Chu (2018) integrated probability, risk, and exposure responses to assess the population size of Chinook salmon. In addition, for experimentally derived data, none of the tests or assays are formally validated or nationally/internationally adopted. However, Moe et al. (2018) performed tests using the aquatic plant *Lemna minor*, which is widely accepted in guidance for toxicity testing (OECD 2006). Nevertheless, as the authors pointed out, *Lemna minor* is used for the analysis of an endpoint, which is the AO in an AOP rather than an entire AOP. Sources of uncertainty were listed by Chu (2018), Moe et al. (2018) and Zgheib et al. (2019), while sensitivity analysis was conducted for all the qAOPs. These types of qAOPs have been modelled using existing software and/or coded in programming languages, i.e. R.

## Overview of mechanistic quantitative linear AOPs and AOP networks

A mechanistic qAOP model is driven by hypothesis testing and utilises a series of deterministic techniques that are discussed briefly below. Ten qAOP models were identified that follow a mechanistic approach, which were assessed in terms of the common features (Table 1) including the additional considerations (Tables 3, 4).

### Problem formulation

The focus of this type of qAOPs relies mainly in understanding the mechanism of toxicity and associated relevant taxonomic domain. The AOs are represented by effects at the ecotoxicological population level, and organ toxicity, e.g. chronic kidney disease, neurodegenerative diseases.

### Mechanistic knowledge and associated data

Five mechanistic AOPs currently available in the AOP-Wiki were quantified, four being endorsed (AOPs IDs 25, 42, 48, 150, 284). Such models have been developed using a variety of types of data including dose- and time-response relationships. For instance, Foran et al. (2019) proposed a modular approach for qAOPs with limited mechanistic data and extensive time required for modelling. The approach focused on making use of the existing information while informing where further tests are needed to provide data for the quantification of all KERs. Some qAOP models have been based on experimental data generated by protocols specifically designed for AOP quantification. For example, to quantify the AOP for developmental neurotoxicity following the inhibition of acetylcholinesterase, Yozzo et al. (2013) studied different levels of biological organisation during zebrafish embryogenesis. Furthermore, in vitro data were employed by the computational model of Zgheib et al. (2019) that quantified the chronic kidney injury in a dose- and time-response manner. qAOP models derived from a combination of both empirical and experimental data will often predict the outcome better and increase the overall confidence in the applicability of the qAOP model. For instance, Muller et al. (2015) described the impact of engineered nanoparticles on hatching of zebrafish eggs using high-throughput data at different timepoints. Model performance showing the experimental differences between the data sources has also been evaluated e.g. Margiotta-Casaluci et al. (2016) investigated in vivo fish egg production following exposure to a chemical class of interest at various concentrations. The final model included data from other studies and the results were compared with human data. At the same time, empirical data are suitable for the optimisation and validation of the predicted response-response relationships as illustrated by Hassan et al. (2017) who optimised the quantification of a classic thyroid hormone (TH) synthesis inhibitor in developmental neurotoxicity in a rodent model using data from the literature. Likewise, Doering et al. (2018) investigated the activation of the aryl hydrocarbon receptor leading to early life stage mortality and validated the resulting qAOP model with empirical evidence. An integration of in silico, in vitro and in vivo data was employed to model the teratogenicity of single and mixture azole fungicides by Battistoni et al. (2019). At the same time, not all quantified AOPs accounted for both dose- and time-scales. Foran et al. (2019) and Doering et al. (2018) focused primarily on predictions based on the tested concentrations. Importantly, most of the published qAOP models utilised linear AOPs, with the exception of Margiotta-Casaluci et al. (2016) who described chronic exposure to synthetic glucocorticoids leading to perturbation in egg production linking three AOPs in a network: disruption of glucose homeostasis, effects on the immune system and androgenic. This integration of evidence shows the complexity of different pathways and their different sensitivities to chemicals.

### Quantitative approaches

Several quantitative approaches were applied for the development of the existing qAOP models. The qAOPs of Muller et al. (2015), Hassan et al. (2017), and Foran et al. (2019) were quantified using purely mathematical equations. Battistoni et al. (2019) developed a multistage dose–response model applying a Bayesian statistical analysis. Besides empirical dose–response, systems biology models were used as a quantitative approach by Battistoni et al. (2019) and Zgheib et al. (2019). Importantly, not all quantified AOPs follow every level of biological organisation. For example, the qAOP formulated by Zgheib et al. (2019) focused on the cellular level due to limited data for the other potential downstream KEs. However, full quantification was undertaken by Muller et al. (2015), Margiotta-Casaluci et al. (2016), Doering et al. (2018), Hassan et al. (2017), Battistoni et al. (2019) who conducted experiments to fill the gaps beyond the available empirical evidence. The qAOP model developed by Conolly et al. (2017) linked multiple models to create a mechanistic qAOP model for aromatase inhibition leading to reproductive dysfunction: a mechanistic hypothalamus–pituitary–gonad model, a vitellogenin liver compartment model, a statistical model relating vitellogenin levels to fecundity and a density-dependent population matrix model. It was later extended from fathead minnow (*Pimephales promelas*) to two other species [female zebrafish (*Danio rerio*) and female Japanese medaka (*Oryzias latipes*)] to broaden the taxonomic domain of applicability and therefore its potential regulatory applications (Doering et al. 2019). Therefore, the AOP ID 25 has three associated qAOP models (Conolly et al. 2017; Doering et al. 2019; Foran et al. 2019).

Regarding the mathematical expressions, linear regression was used by Doering et al. (2018) and Foran et al. (2019), while exponential equations were used by Foran et al. (2019) and by Hassan et al. (2017) for the computational prediction of thyroid hormone disruption on the developing brain in rats. Elsewhere, Battistoni et al. (2019) used kinetic equations adapted from a published systems biology mathematical model to simulate the kinetics of single chemicals and mixtures and the perturbation which may lead the co-exposure of chemicals. A systems biology model was also employed by Zgheib et al. (2019) that used over 50 differential equations and, as a result, showed the need of extensive parametrisation (335 parameters). A combination of linear models, kinetic equations and statistical analysis was considered by Muller et al. (2015) in a study of copper nanoparticles. The qAOP models of Margiotta-Casaluci et al. (2016) and Yozzo et al. (2013) applied statistical analysis, i.e. one-way analysis of variance (ANOVA) to the experiments conducted to evaluate the pathway of interest quantitatively.

### Regulatory applicability

All qAOPs have applications in ecological risk assessment, while the qAOP model developed by Foran et al. (2019) is intended for screening and/or prioritisation purposes and that developed by Zgheib et al. (2019) is proposed for human health risk assessment. The qAOP of Conolly et al. (2017) showed additional potential applications: comparing the qAOP simulations to empirical data, how a response-response function can be derived and how to estimate the benchmark dose for an untested chemical using toxicity equivalent factor.

### Additional considerations

The adjacency and non-adjacency of KERs was considered by Hassan et al. (2017), Doering et al. (2018) and Foran et al. (2019). Hassan et al. (2017) developed the non-adjacent KER using literature data to model the gaps. Doering et al. (2018) used non-adjacent KERs to check and verify the linkage between KEs and the AO. Foran et al. (2019) proposed a modular approach as a feasible solution to the AOPs lacking empirical dose- and time-response data. Zgheib et al. (2019) used a mathematical inversion technique to derive chemical-independent KERs from a series of dose–time–response relationships. Four qAOPs incorporated kinetics: Battistoni et al. (2019), Hassan et al. (2017), Margiotta-Casaluci et al. (2016) and Muller et al. (2015). Furthermore, Battistoni et al. (2019) included a modulating factor, i.e. identifying that ethanol can also inhibit retinoic acid synthesis, and a negative feedback loop, i.e. regulation of retinoic acid resulting from increased synthesis of CYP26A1. Doering et al. (2018, 2019) developed a qAOP that is applicable across species. The uncertainty of the model was considered by Hassan et al. (2017), Doering et al. (2018), Battistoni et al. (2019) and Foran et al. (2019). Sensitivity analysis was performed by Margiotta-Casaluci et al. (2016) and Zgheib et al. (2019). The mathematical equations and/or the code of the qAOP models of Hassan et al. (2017), Doering et al. (2018), Zgheib et al. (2019) and Muller et al. (2015) are accessible.

## Software tools

A variety of software tools used for the development of the qAOPs were identified in this study (Supplementary Information Table S2). In total, 20 tools were distinguished, with 11 of them being publicly available. The range of software tools can be classified into tools used for (i) data analysis, (ii) modelling, simulation and calibration, and (iii) model storage. The most commonly used tools were Microsoft Excel, the *drc* R package for writing the mathematical functions of dose responses, MC Sim for statistical analysis, and BayesiaLab for probabilistic modelling. A unique tool is the Bayesian Inference for Substance and Chemical Toxicity (BISCT) software developed specifically to predict quantitative estimates based on the toxicological evidence. Another important tool used is Effectopedia, an open platform that allows qAOP models to be stored in a central location. This compilation of software shows the huge potential in the development of appropriate tools to help advance and apply the qAOP concept.

## Conclusions and future directions

This review has summarised the recent progress made in the development of qAOP models. A list of common features typically used when developing qAOP models has been identified, namely problem formulation, mechanistic knowledge and associated data, quantitative approaches, and additional considerations derived from published definitions in the scientific literature. Hence, following the conceptual classes of qAOP models proposed by Gust et al. (2016) and Perkins et al. (2019b), existing qAOPs were identified and characterised according to the predefined common features. The qAOPs discussed illustrate a range of computational techniques and software tools applicable to such modelling. Importantly, these examples highlight the powerful capability of a qAOP model to integrate diverse types of data (physico-chemical, in silico, in vitro, in vivo).

There is currently no guidance on how to develop and evaluate qAOP models for regulatory applications. As more examples of qAOPs become available, there will be an increasing need to provide a coherent framework to support the evaluation and purpose-specific application of qAOPs in a regulatory context. While it is beyond the scope of this review to outline such a framework, a number of elements (principles) can be identified, some of which may be essential, and others desirable, depending on the application.

An ideal qAOP should:

- Predict a defined AO (defined endpoint);
- Address a specified regulatory question and context of use (problem formulation);
- Be consistent with the qualitative description of the AOP of interest;
- Have a clear domain of applicability (including species, taxa, modulating factors);
- Be characterised in terms of its predictive performance and robustness (uncertainty and sensitivity analysis);
- Be transparent and traceable, to allow independent evaluation and verification of the qAOP model (including input data, simulated outputs, and correct implementation of the mathematical equations);
- Be understandable and user-friendly, to ease its interpretation and application;
- Be flexible, to allow analysis of both existing and new molecules;
- Be updateable, to refine parameter estimates by incorporating new data as they become available (in such cases, versioning of the qAOP model will be required);
- Be reproducible, to enhance the confidence in the consistency and accuracy of the qAOP model output;
- Be portable, so that the qAOP model can be integrated with other mathematical models, such as kinetic models;
- Be publicly available, either in the form of a working platform, or availability of code.

Although current efforts in qAOP modelling are limited, the field is gaining momentum. This review can therefore serve as a starting point to formulate formal guidance on the development, assessment and application of probabilistic and mechanistic qAOPs in chemical risk assessment. Future work should consider best practices and provide examples of tackling the challenges in developing qAOP models.

## Electronic supplementary material

Below is the link to the electronic supplementary material.

Supplementary file1 (DOCX 99 kb)
