# Supplementary material for: Quantitative adverse outcome pathway (qAOP) models for toxicity prediction
Source: Arch Toxicol. 2020 May 18;94(5):1497–510. doi: 10.1007/s00204-020-02774-7 (PMC7261727; doi:10.1007/s00204-020-02774-7)
Supplement: Supplementary file 1 — Supplementary file1 (DOCX 99 kb) [file 204_2020_2774_MOESM1_ESM.docx]

Supplementary Information

Archives of Toxicology

**Quantitative Adverse Outcome Pathway (qAOP) Models for Toxicity Prediction**

Nicoleta Spinu^1^, Mark T.D. Cronin^1^, Steven J. Enoch^1^, Judith C. Madden^1^, Andrew P. Worth^2^

^1^School of Pharmacy and Biomolecular Sciences, Liverpool John Moores University, Byrom Street, Liverpool,
L3 3AF, UK

^2^European Commission, Joint Research Centre (JRC), Ispra, Italy

Corresponding Author: Andrew P. Worth; Tel: +39 0332 789566; E-mail: [andrew.worth@ec.europa.eu](mailto:andrew.worth@ec.europa.eu); European Commission, Joint Research Centre (JRC), Ispra, Italy

**Supplementary Tables**

[**Table S1.** Overview of 23 definitions for the quantitative Adverse Outcome Pathway concept in chronological order, the common features of a qAOP model derived from the highlighted key words, and the type of qAOP (quantitative weight of evidence, probabilistic or mechanistic) implied by the definition. 2](#_Toc38534596)

[**Table S2.** List of software used for modelling probabilistic and/or mechanistic qAOPs. 8](#_Toc38534597)

**Table S1.** Overview of 23 definitions for the quantitative Adverse Outcome Pathway concept in chronological order, the common features of a qAOP model derived from the highlighted key words, and the type of qAOP (quantitative weight of evidence, probabilistic or mechanistic) implied by the definition.

| Common features | Type of qAOP | Extracted qAOP definition |
| --- | --- | --- |
| Mechanistic knowledge and associated data  Quantitative approaches  Additional considerations | Mechanistic | *“For maximum application across levels of biological organization, AOPs should encompass* ***test endpoints or nodes*** *that can be quantitatively related to demographic traits of the model* ***population****. Models that describe* ***lower-level*** *mechanistic detail be coupled with those being used for modeling population processes and necessitates* ***empirically*** *derived* ***quantitative relationships*** *between the individual endpoint and inputs to the demographic rates of the population models. Establishing these relationships often requires simultaneous bottom-up and top-down* ***integration*** *of the physiology of the organism with the natural history of the population.”*  (Kramer et al. 2011) |
| Quantitative approaches  Additional considerations | Mechanistic | *“Ideally, causality across AOPs is approached not only in a qualitative, but also in a* ***quantitative*** *way relating* ***exposure*** *to the adverse outcome… The situation is relatively straightforward if the extent to which a MIE or KE is altered and is known to be sufficient to trigger the final AO. It is assumed that the AO occurs only after a biologically meaningful* ***overall threshold*** *has been passed.”* (Bal-Price et al. 2015) |
| Quantitative approaches  Regulatory applicability  Additional considerations | Mechanistic | *“The use of AOPs in a* ***full risk assessment*** *would require a* ***quantitative*** *description of the links between* ***suborganismal*** *changes, ecologically relevant outcomes in* ***individuals*** *and* ***population****-level responses. Moreover, a complete risk assessment would need to be based not only on the simplified* ***toxicodynamic*** *sequence of events depicted in the AOP itself, but also take into account* ***chemical****- (e.g. external and internal exposure) and* ***situation****-specific (e.g. outcomes for a specific field population) aspects.* ***Bioavailability*** *and* ***toxicokinetic*** *processes require specific attention if the AOP is to be used for any quantitative assessments with* ***regulatory relevance****. Furthermore, to enable AOP application for quantitative risk assessment, the linkages between KEs and AOs need to be defined quantitatively. To establish a quantitative AOP, thresholds for upstream MIE or KE to trigger the downstream KEs or AOs need to be defined, taking into account the potential* ***modifying*** ***factors*** *as well as site-specific contexts to the fullest extent possible.”* (Groh et al. 2015) |
| Quantitative approaches  Regulatory applicability  Additional considerations | Mechanistic | *“A further academic perspective for future AOP research, with a strong link to* ***regulatory applications****, is to* ***quantitatively*** *describe the* ***causal*** ***links*** *of the AOP chain to facilitate the prediction of AOs based on the analysis of molecular initiating events or key events and the* ***extrapolation*** *between models and species. For a full quantitative approach, established* ***toxicokinetic*** *and* ***toxicodynamic*** *models could be applied to effectively integrate* ***exposure*** *and toxicokinetic information.”* (Groh and Tollefsen 2015) |
| Mechanistic knowledge and associated data  Quantitative approaches  Regulatory applicability | Mechanistic | *“The need for predictive power has been a strong impetus to develop adverse outcome pathway (AOP) analyses, which describe networks of causally linked events at diﬀerent levels of biological organization, and to develop* ***general quantitative methods*** *that summarize toxicant impact in process-based toxicity measures.* ***Integration*** *of these two developments into* ***quantitative AOP approaches*** *promises to yield powerful* ***predictive tools*** *for* ***ecological risk assessment****. These approaches, in which toxicity metrics relate to chemical, biological and ecological processes, eliminate or reduce the dependence of toxicity assessments on experimental design, choices of endpoint, and species of organism and chemical compound. Furthermore, quantitative AOPs open the way to using results from* ***(semi)automated high-throughput*** *and* ***high content screening tests*** *to anticipate the impact of toxicants on processes at ecologically relevant levels of biological organization. Typically, those rapid and cost-eﬃcient screening tests record molecular, cellular or individual responses to toxicant exposure in a* ***dose−response manner*** *in order to* ***rank the hazard*** *of a group of compounds.* ***Data*** *from those screening tests could also be analyzed within a quantitative AOP framework.”* (Muller et al. 2015) |
| Quantitative approaches | Probabilistic and mechanistic | *“In an idealized case, an AOP would include a description of all key events, delineation of methods which can be used to measure each key event, descriptions of each key event relationship (KER), and* ***quantitative models*** *for each KER to permit* ***statistical prediction*** *of a downstream key event from an upstream key event. If all of this information were available, quantitative predictions of the adverse outcome (AO) could be made from an upstream key event.”* (Patlewicz et al. 2015) |
| Quantitative approaches  Additional considerations | Mechanistic | *“Stronger scientific confidence in KE relationships are required to enable AOPs to be used to understand fully pathway homology* ***across species****. They permit development of* ***quantitative models*** *for* ***extrapolating*** *difficult to measure outcomes such as* ***population*** *level effects from KEs.”*  (Perkins et al. 2015) |
| Quantitative approaches  Regulatory applicability  Additional considerations | Quantitative weight of evidence and mechanistic | *“Semiquantitative and quantitative AOPs (qAOPs) have been targeted for development to serve as* ***predictive models*** *in the quantitative toolbox for human and environmental health* ***impact assessment****. To enable transition of AOPs to* ***life cycle impact assessment*** *(LCIA), AOPs must become more quantitative in nature, with specific emphasis on establishing* ***quantitative dose–response relationships*** *between MIEs and various KEs to the adverse outcome of concern. Therefore, to facilitate LCIA, the underlying AOP framework must be quantitative and able to predict dose–response relationships between activation of MIEs or other key events to adverse outcomes that are directly involved in fitness (i.e.,* ***effects*** *that impair the ability of individuals to survive or reproduce). A quantitative AOP should provide numerically based assessments to attempt to* ***quantify weight of evidence*** *(WoE).”* (Gust et al. 2016) |
| Regulatory applicability | Mechanistic | *“To provide information useful for* ***chemical risk assessment****, AOPs need to have some quantitative information relevant to important KERs. Beyond characterizing the pathway, it is important to understand the* ***dose*** *that activates the pathway, and if it is* ***relevant*** *to human or ecological exposure scenarios.”* (Kleinstreuer et al. 2016) |
| Mechanistic knowledge and associated data | Mechanistic | *“It is important to understand the key event relationships (KERs) and to* ***provide*** ***relevant*** ***information*** *or, even better,* ***quantitative data*** *supporting KERs, especially between the early KEs.”* (Bal-Price et al. 2017) |
| Mechanistic knowledge and associated data  Quantitative approaches  Regulatory applicability  Additional considerations | Probabilistic and mechanistic | *“The term quantitative AOP (qAOP) refers to a loosely defined, but relatively advanced stage in the progression of AOP development and description. At this stage,* ***quantitative understanding*** *of the relationships underlying transition from one KE to the next, as well as* ***critical factors*** ***that can modulate*** *those relationships, are sufficiently well-defined to allow* ***quantitative*** ***prediction*** *of the* ***probability*** *or* ***severity*** *of the AO occurring for a given activation of the MIE.* ***Information*** *concerning the quantitative understanding of what defines the transition from one KE in an AOP to the next is thus captured and included (where possible) in the KE relationship descriptions. That quantitative understanding may take* ***many forms****, depending on the extent of the* ***available, relevant data****. In the case of a relatively limited data set containing little or no or dose−response and time-course information, the relationship between adjacent KEs may be as simple as a* ***linear regression equation*** *linking an upstream with an immediately downstream KE. With richer data sets, reflecting fuller dose-response and time-course designs, the quantitative understanding may be encoded into sophisticated* ***biologically based models*** *that simulate complex, nonlinear, dynamics that can result from* ***feedback*** ***loops****,* ***adaptive*** *and* ***compensatory responses****,* ***stochastic influences****,* ***interactions*** *with other pathways, and/or influences of external or internal* ***modulating factors****. Whatever form they take, quantitative understanding of the KE relationships encompassed in an AOP description can facilitate a broader spectrum of* ***applications****. Consequently, there is interest in developing the quantitative understanding and description of AOPs to the extent that* ***regulatory*** ***needs*** *warrant and resources allow.”* (Conolly et al. 2017) |
| Regulatory applicability | Quantitative weight of evidence and mechanistic | *“KERs facilitate inference or extrapolation based on the premise that if the upstream KE is altered to a sufficient degree, predictable changes (qualitative or quantitative) can be expected in the downstream event in the hypothesized AOP. For empirical support, qualitative consideration of the extent of supporting data or* ***WOE*** *for hypothesized AOPs takes into account “patterns” of quantitative relationships for KERs (i.e., the extent to which* ***temporal and dose response*** *patterns align with what would be anticipated, for essential key events in an AOP). This differs from quantitation of the KERs, addressing essentially how much change in KEup is needed to evoke some unit of change in KEdown as a basis for developing* ***predictive response models****.”* (Meek 2017) |
| Mechanistic knowledge and associated data  Quantitative approaches | Mechanistic | *“Currently there are few, or no, examples of* ***(Q)SAR*** *or* ***QAAR*** *models for Key Event Relationships, although some* ***in silico models*** *for Key Event Relationships are becoming available, especially in the form of quantitative AOPs (qAOPs). Quantitative models for the MIE, as well as for Key Events and Key Event Relationships require a more* ***complete data set*** *with information from a greater number of* ***compounds*** *covering a range of* ***activity*** *and* ***properties****.”* (Cronin and Richarz 2017) |
| Quantitative approaches  Regulatory applicability  Additional considerations | Mechanistic | ***“Computational predictive modelling*** *can be applied to quantitatively describe the sequences of key events (KEs) and their relationships (KE relationships, KER) and the biologic processes of pathogenesis that comprise an AOP. The* ***reliability*** *of predictions from these models is improved with greater* ***understanding of the biologic foundation*** *of the AOP. Predictions also are improved by complete appreciation of the* ***quantitative determinants*** *from upstream biologic perturbations to subsequent downstream overt organismal effects comprising the AO. When* ***mathematical*** *descriptions of these relationships are biologically driven* ***equations****, a mechanistic quantitative AOP (qAOP) is derived.”* (Hassan et al. 2017) |
| Mechanistic knowledge and associated data  Quantitative approaches  Regulatory applicability | Probabilistic and mechanistic | *“Information captured in the “quantitative understanding of the linkage” section of the KER descriptions within the AOP framework provides the foundation for addressing this desire for quantification. Quantitative AOPs can be described in various ways, ranging from* ***expert judgment-based scoring****, requiring limited information, where elements of the AOP are weighted using expert opinion, to more* ***probabilistic approaches****, where statistical relationships exist between the MIE/KE and the adverse outcome, to* ***mechanistic approaches****. The more mechanistic approaches employ* ***mathematical models*** *or relationships of MIE, KE, and KER (e.g., response–response relationships between KERs) to quantitatively* ***predict the risk*** *of an adverse effect given specified* ***initial conditions*** *(e.g., a set of exposure conditions).”* (LaLone et al. 2017) |
| Quantitative approaches | Mechanistic | *“Whereas an AOP description lays out the sign posts within a biological system that indicate progression toward an AO, computational models can quantitatively* ***simulate*** *the* ***dynamics*** *of the complex biology at multiple scales that dictate dose–response and time–course behaviors and* ***define the conditions*** *under which perturbation of early KEs in the pathway will ultimately lead to the AO, or not.”* (Wittwehr et al. 2017) |
| Quantitative approaches  Additional considerations | Mechanistic | *“It is possible to assemble quantitative AOPs (qAOPs) that consider* ***quantitative relationships*** *between KEs, including* ***feedback*** *models* ***designed*** *to reflect system regulation, to predict* ***AOs****.”* (Ankley and Edwards 2018) |
| Mechanistic knowledge and associated data  Quantitative approaches  Additional considerations | Mechanistic | *“The* ***integration*** *of all* ***information*** *will also lead to the development of quantitative AOPs that can be used for* ***dose-response analyses****, and iteratively, inform* ***reﬁnements*** *of the next generation of mechanistic IATAs.”* (Clippinger et al. 2018) |
| Mechanistic knowledge and associated data  Quantitative approaches  Regulatory applicability  Additional considerations | Probabilistic and mechanistic | *“The most advanced developments in AOPs, known as quantitative AOPs (qAOPs), have potential utility to* ***quantitative ecological risk assessments****. A qAOP describes* ***quantitative response−response relationships*** *linking the molecular initiating event and adverse outcome to enable quantitative prediction of the* ***probability*** *of occurrence or* ***severity*** *of an adverse outcome for a given magnitude of chemical interaction with a molecular initiating event. Depending upon the extent of* ***mechanistic understanding*** *and the needs in terms of* ***regulatory application****, a qAOP could be as simple as a* ***linear regression*** *that quantitatively links the molecular initiating event to the adverse outcome, or as complex as a consecutive series of* ***nonlinear models*** *which describe responses at several levels of biological organization and* ***simulate*** *associated internal and external* ***modifying*** ***factors****.”* (Doering et al. 2018b) |
| Mechanistic knowledge and associated data  Quantitative approaches | Mechanistic | *“A quantitative adverse outcome pathway (qAOP) is a* ***mathematical/computational model*** *that represents the* ***dynamic*** *processes linking a molecular initiating event with an adverse outcome. A unique feature that distinguishes a qAOP from other biologically based mathematical models is the prediction of key events that are part of the qualitative adverse outcome pathway and are* ***measurable*** ***experimentally****.”*  (Schultz and Watanabe 2018) |
| Additional considerations | Mechanistic | *“Quantitative AOPs will help answer what* ***level of in vitro perturbation*** *should be used as a point of departure (PoD) for quantitative in vitro to in vivo extrapolations (QIVIVE).”* (Beilmann et al. 2018) |
| Quantitative approaches | Probabilistic and mechanistic | *“For each pair of KEs, a quantitative KE relationship (KER) can be derived as a* ***response-response function*** *or a* ***conditional probability matrix*** *describing the anticipated change in a KE based on the response of the prior KE. This transfer of response across KERs can be used to assemble a quantitative AOP*.” (Foran et al. 2019) |
| Mechanistic knowledge and associated data  Quantitative approaches  Regulatory applicability  Additional considerations | Probabilistic and mechanistic | *“Quantitatively, a KER may be defined in terms of* ***regressions*** *between KEs response-response relationships or dose-dependent transitions. They may take the form of* ***simple mathematical equations*** *or sophisticated* ***biologically based computational models*** *that consider other* ***modulating factors****, such as compensatory responses, or interactions with other biological or environmental variables. Depending on the level and nature of* ***empirical data available****, there is a continuum of AOPs from purely descriptive qualitative AOPs to qAOP models with detailed response-response relationships that allow one to infer the* ***magnitude*** *or* ***probability*** *of an AO. Here, we define a full qAOP model to be any mathematical construct that models the dose response or response-response relationships of all KERs described in an AOP, a partial qAOP as a construct that models the dose/response-response relationships of more than one KER, and a quantitative KER as a construct that models a single dose/response-response relationship. qAOP models support explicit* ***incorporation*** *of complex relationships, such as* ***feedback loops****,* ***thresholds****, and* ***signaling cascades*** *that are generally embedded in the KE or KER of descriptive AOPs. Models incorporating complex biological relationships can create predictions with greater biological fidelity to* ***support hazard and risk assessment*** *than models with simplified assumptions.”* (Perkins et al. 2019) |

**Table S2.** List of software used for modelling probabilistic and/or mechanistic qAOPs.

| Tool Name | Functionality | URL | Use Rights | qAOP Examples |
| --- | --- | --- | --- | --- |
| BayesiaLab | Model generation, analysis, simulation, and optimisation | <https://www.bayesialab.com/> | Restricted by licence | Carriger et al. (2016); Jaworska et al. (2015) |
| BISCT | Prediction of an adverse event likely to occur given the evidence | <https://github.com/DataSciBurgoon/bisct> | Open access | Perkins et al. (2019) |
| *bootstrap* R  package | Bootstrap, cross-validation, jackknife | <https://cran.r-project.org/web/packages/bootstrap/> | Open access | Jeong et al. (2018) |
| *drc* R package | Analysis of dose-response data | <https://cran.r-project.org/web/packages/drc/index.html> | Open access | Chu (2018); Moe et al. (2018) |
| Effectopedia | Storage of a qAOP model | <https://www.effectopedia.org> | Open access | Zgheib et al. (2019) |
| lmtest | A collection of tests, data sets, and examples for diagnostic checking in linear regression models | <https://cran.r-project.org/web/packages/lmtest/index.html> | Open access | Chu (2018) |
| MC Sim | Bayesian statistical inference | <https://www.gnu.org/software/mcsim/> | Open access | Battistoni et al. (2019); Hack et al. (2010); Zgheib et al. (2019) |
| MC Stan | Model generation, simulation, calibration | <https://mc-stan.org/> | Open access | Zgheib et al. (2019) |
| Microsoft Excel | Statistical analysis | <https://products.office.com/en-us/excel> | Restricted by licence | Foran et al. (2019); Hack et al. (2010); Hassan et al. (2017) |
| Netica | Model construction, optimisation | <https://www.norsys.com/download.html> | Restricted by licence | Chu (2018) |
| *PerformanceAnalytics R package* | Correlation analysis | <https://cran.r-project.org/web/packages/PerformanceAnalytics/index.html> | Open access | Jeong et al. (2018) |
| Samlam | BN modelling and reasoning | <http://reasoning.cs.ucla.edu/samiam/> | Open access | Jeong et al. (2018) |
| *SigmaPlot* | Graphs plotting | <http://sigmaplot.co.uk/products/sigmaplot/sigmaplot-details.php> | Restricted by licence | Jeong et al. (2018) |
| SPSS | Statistical analysis | <https://www.ibm.com/uk-en/products/spss-statistics> | Restricted by licence | Jeong et al. (2018); Yozzo et al. (2013) |
| WEKA | Collection of machine learning algorithms for data mining tasks | <https://www.cs.waikato.ac.nz/~ml/weka/index.html> | Open access | Furxhi et al. (2019) |

**(Continued)**

**Table S2.** Continued

| Tool Name | Functionality | URL | Use Rights | qAOP Examples |
| --- | --- | --- | --- | --- |
| US EPA Benchmark Dose Software | Analysis of dichotomous (quantal) data, continuous data, nested developmental toxicology data, and multiple tumour analysis | <https://www.epa.gov/bmds/benchmark-dose-software-bmds-version-311-download> | Open access | Furxhi et al. (2019) |
| GraphPad Prism | Analysis and plotting the data | <https://www.graphpad.com/scientific-software/prism/> | Restricted by licence | Doering et al. (2018a) |
| Matlab | Analysis and design processes | <https://www.mathworks.com/products/matlab.html> | Restricted by licence | Hassan et al. (2017) |
| SAS software | Statistical analyses | <https://www.sas.com/en_us/software/sas9.html> | Restricted by licence | Hassan et al. (2017) |
| SigmaStat software | Statistical analyses | <https://systatsoftware.com/products/sigmastat/> | Restricted by licence | Margiotta-Casaluci et al. (2016) |

# References

Ankley GT, Edwards SW (2018) The adverse outcome pathway: A multifaceted framework supporting 21(st) century toxicology. Curr Opin Toxicol 9:1-7 doi:10.1016/j.cotox.2018.03.004

Bal-Price A, Crofton KM, Leist M, et al. (2015) International STakeholder NETwork (ISTNET): creating a developmental neurotoxicity (DNT) testing road map for regulatory purposes. Arch Toxicol 89(2):269-287 doi:10.1007/s00204-015-1464-2

Bal-Price A, Lein PJ, Keil KP, et al. (2017) Developing and applying the adverse outcome pathway concept for understanding and predicting neurotoxicity. Neurotoxicology 59:240-255 doi:10.1016/j.neuro.2016.05.010

Battistoni M, Di Renzo F, Menegola E, Bois FY (2019) Quantitative AOP based teratogenicity prediction for mixtures of azole fungicides. Comput Toxicol(11):72-81 doi:<https://doi.org/10.1016/j.comtox.2019.03.004>

Beilmann M, Boonen H, Czich A, et al. (2018) Optimizing drug discovery by investigative toxicology: Current and future trends. ALTEX 36(2):289-313 doi:10.14573/altex.1808181

Carriger JF, Martin TM, Barron MG (2016) A Bayesian network model for predicting aquatic toxicity mode of action using two dimensional theoretical molecular descriptors. Aquat Toxicol 180:11-24 doi:<https://doi.org/10.1016/j.aquatox.2016.09.006>

Chu VR (2018) Assessing the effects of chemical mixtures using a Bayesian Network-Relative Risk Model (BNRRM) integrating Adverse Outcome Pathways (AOPs) in four watersheds. WWU Graduate School Collection 699 doi:<https://cedar.wwu.edu/wwuet/699>

Clippinger AJ, Allen D, Behrsing H, et al. (2018) Pathway-based predictive approaches for non-animal assessment of acute inhalation toxicity. Toxicol in Vitro 52:131-145 doi:<https://doi.org/10.1016/j.tiv.2018.06.009>

Conolly RB, Ankley GT, Cheng W, et al. (2017) Quantitative adverse outcome pathways and their application to predictive toxicology. Environ Sci Technol 51(8):4661-4672 doi:<https://doi.org/10.1021/acs.est.6b06230>

Cronin M, Richarz A-N (2017) Relationship between adverse outcome pathways and chemistry-based in silico models to predict toxicity. Appl Vitr Toxicol 3(4):286–297 doi:10.1089/aivt.2017.0021

Doering JA, Wiseman S, Giesy JP, Hecker M (2018a) A cross-species quantitative adverse outcome pathway for activation of the aryl hydrocarbon receptor leading to early life stage mortality in birds and fishes. Environ Sci tech 52(13):7524-7533 doi:10.1021/acs.est.8b01438

Doering JA, Wiseman S, Giesy JP, Hecker M (2018b) A cross-species quantitative adverse outcome pathway for activation of the aryl hydrocarbon receptor leading to early life stage mortality in birds and fishes. Environ Sci Technol 52(13):7524-7533 doi:<https://doi.org/10.1021/acs.est.8b01438>

Foran CM, Rycroft T, Keisler J, Perkins EJ, Linkov I, Garcia-Reyero N (2019) A modular approach for assembly of quantitative adverse outcome pathways. ALTEX 36(3):353-362 doi:<https://doi.org/10.14573/altex.1810181>

Furxhi I, Murphy F, Poland CA, Sheehan B, Mullins M, Mantecca P (2019) Application of Bayesian networks in determining nanoparticle-induced cellular outcomes using transcriptomics. Nanotoxicology 13(6):827-848 doi:<https://doi.org/10.1080/17435390.2019.1595206>

Groh KJ, Carvalho RN, Chipman JK, et al. (2015) Development and application of the adverse outcome pathway framework for understanding and predicting chronic toxicity: I. Challenges and research needs in ecotoxicology. Chemosphere 120:764-777 doi:10.1016/j.chemosphere.2014.09.068

Groh KJ, Tollefsen KE (2015) The Challenge: Adverse outcome pathways in research and regulation-Current status and future perspectives. Environ Toxicol Chem 34(9):1935 doi:10.1002/etc.3042

Gust KA, Collier ZA, Mayo ML, Stanley JK, Gong P, Chappell MA (2016) Limitations of toxicity characterization in life cycle assessment: Can adverse outcome pathways provide a new foundation? Integr Environ Assess Manag 12(3):580-90 doi:<https://doi.org/10.1002/ieam.1708>

Hack CE, Haber LT, Maier A, et al. (2010) A Bayesian network model for biomarker-based dose response. Risk Anal 30(7):1037-1051 doi:<https://doi.org/10.1111/j.1539-6924.2010.01413.x>

Hassan I, El-Masri H, Kosian PA, Ford J, Degitz SJ, Gilbert ME (2017) Neurodevelopment and thyroid hormone synthesis inhibition in the rat: quantitative understanding within the adverse outcome pathway framework. Toxicol Sci 160(1):57-73 doi:<https://doi.org/10.1093/toxsci/kfx163>

Jaworska JS, Natsch A, Ryan C, Strickland J, Ashikaga T, Miyazawa M (2015) Bayesian integrated testing strategy (ITS) for skin sensitization potency assessment: a decision support system for quantitative weight of evidence and adaptive testing strategy. Arch Toxicol 89(12):2355-2383 doi:<https://doi.org/10.1007/s00204-015-1634-2>

Jeong J, Song T, Chatterjee N, Choi I, Cha YK, Choi J (2018) Developing adverse outcome pathways on silver nanoparticle-induced reproductive toxicity via oxidative stress in the nematode Caenorhabditis elegans using a Bayesian network model. Nanotoxicology 12(10):1182-1197 doi:<https://doi.org/10.1080/17435390.2018.1529835>

Kleinstreuer NC, Sullivan K, Allen D, et al. (2016) Adverse outcome pathways: From research to regulation scientific workshop report. Regul Toxicol Pharmacol 76:39-50 doi:10.1016/j.yrtph.2016.01.007

LaLone CA, Ankley GT, Belanger SE, et al. (2017) Advancing the adverse outcome pathway framework-An international horizon scanning approach. Environ Toxicol Chem 36(6):1411-1421 doi:10.1002/etc.3805

Margiotta-Casaluci L, Owen SF, Huerta B, et al. (2016) Internal exposure dynamics drive the adverse outcome pathways of synthetic glucocorticoids in fish. Sci Rep 6:21978 doi:<https://doi.org/10.1038/srep21978>

Meek MEB (2017) AOPs in hazard characterization for human health. Curr Opin Toxicol 3:80-86 doi:10.1016/j.cotox.2017.06.002

Moe J, Wayne L, Xie L, Tollefsen KE, Kotamäki N (2018) Quantification of an adverse outcome pathway by Bayesian network modelling: extrapolation from molecular events to demographic responses in Lemna minor. Paper presented at the SETAC Europe 13th Special Science Symposium, Brussels, Belgium, https://sesss13.setac.org/wp-content/uploads/2018/11/P15.pdf

Muller EB, Lin S, Nisbet RM (2015) Quantitative adverse outcome pathway analysis of hatching in zebrafish with CuO nanoparticles. Environ Sci Technol 49(19):11817-11824 doi:<https://doi.org/10.1021/acs.est.5b01837>

Patlewicz G, Simon TW, Rowlands JC, Budinsky RA, Becker RA (2015) Proposing a scientific confidence framework to help support the application of adverse outcome pathways for regulatory purposes. Regul Toxicol Pharm 71(3):463-477 doi:10.1016/j.yrtph.2015.02.011

Perkins EJ, Antczak P, Burgoon L, et al. (2015) Adverse outcome pathways for regulatory applications: examination of four ase studies with different degrees of completeness and scientific confidence. Toxicol Sci 148(1):14-25 doi:<https://doi.org/10.1093/toxsci/kfv181>

Perkins EJ, Ashauer R, Burgoon L, et al. (2019) Building and applying quantitative adverse outcome pathway models for chemical hazard and risk assessment. Environ Toxicol Chem 38(9):1850-1865 doi:<https://doi.org/10.1002/etc.4505>

Schultz IR, Watanabe KH (2018) The Development of Quantitative AOPs. In: Garcia-Reyero N, Murphy CA (eds) A systems biology approach to advancing adverse outcome pathways for risk assessment. 1 edn. Springer International Publishing, Cham, p 263-280

Wittwehr C, Aladjov H, Ankley G, et al. (2017) How adverse outcome pathways can aid the development and use of computational prediction models for regulatory toxicology. Toxicol Sci 155(2):326-336 doi:<https://doi.org/10.1093/toxsci/kfw207>

Yozzo KL, McGee SP, Volz DC (2013) Adverse outcome pathways during zebrafish embryogenesis: a case study with paraoxon. Aquat Toxicol 126:346-354 doi:<https://doi.org/10.1016/j.aquatox.2012.09.008>

Zgheib E, Ga W, Limonciel A, et al. (2019) Application of three approaches for quantitative AOP development to renal toxicity. Comput Toxicol(11):1-13 doi:10.1016/j.comtox.2019.02.001
